# Propylthiouracil Is Teratogenic in Murine Embryos

**DOI:** 10.1371/journal.pone.0035213

**Published:** 2012-04-18

**Authors:** Valeria C. Benavides, Murali K. Mallela, Carmen J. Booth, Christopher C. Wendler, Scott A. Rivkees

**Affiliations:** 1 Section of Developmental Endocrinology and Biology, Yale Pediatric Thyroid Center, Department of Pediatrics, Yale University School of Medicine, New Haven, Connecticut, United States of America; 2 Section of Comparative Medicine, Yale University School of Medicine, New Haven, Connecticut, United States of America; 3 Department of Pediatrics, University of Florida, Gainesville, Florida, United States of America; Fudan University, China

## Abstract

**Background:**

Hyperthyroidism during pregnancy is treated with the antithyroid drugs (ATD) propylthiouracil (PTU) and methimazole (MMI). PTU currently is recommended as the drug of choice during early pregnancy. Yet, despite widespread ATD use in pregnancy, formal studies of ATD teratogenic effects have not been performed.

**Methods:**

We examined the teratogenic effects of PTU and MMI during embryogenesis in mice. To span different periods of embryogenesis, dams were treated with compounds or vehicle daily from embryonic day (E) 7.5 to 9.5 or from E3.5 to E7.5. Embryos were examined for gross malformations at E10.5 or E18.5 followed by histological and micro-CT analysis. Influences of PTU on gene expression levels were examined by RNA microarray analysis.

**Results:**

When dams were treated from E7.5 to E9.5 with PTU, neural tube and cardiac abnormalities were observed at E10.5. Cranial neural tube defects were significantly more common among the PTU-exposed embryos than those exposed to MMI or vehicle. Blood in the pericardial sac, which is a feature indicative of abnormal cardiac function and/or abnormal vasculature, was observed more frequently in PTU-treated than MMI-treated or vehicle-treated embryos. Following PTU treatment, a total of 134 differentially expressed genes were identified. Disrupted genetic pathways were those associated with cytoskeleton remodeling and keratin filaments. At E 18.5, no gross malformations were evident in either ATD group, but the number of viable PTU embryos per dam at E18.5 was significantly lower from those at E10.5, indicating loss of malformed embryos. These data show that PTU exposure during embryogenesis is associated with delayed neural tube closure and cardiac abnormalities. In contrast, we did not observe structural or cardiac defects associated with MMI exposure except at the higher dose. We find that PTU exposure during embryogenesis is associated with fetal loss. These observations suggest that PTU has teratogenic potential.

## Introduction

Graves' disease (GD) is caused by activating autoantibodies to the thyrotropin (TSH) receptor (TSHR-Ab) leading to excessive thyroid hormone secretion and thyromegaly [1], [2], [3]. GD is the most common cause of hyperthyroidism during pregnancy and is estimated to affect 1 in 500 to 1,000 women [4]. Considering that there are about 4 million births per year in the United States (US) [5], 4,000 to 8,000 women per year will have GD during pregnancy in the US [4], [6].

Under- or untreated hyperthyroidism during pregnancy is associated with an increased risk of miscarriage, premature birth, intrauterine growth retardation, fetal demise, maternal hypertension, and thyroid storm [1], [6], [7]. As such, optimal treatment of GD during pregnancy is essential for favorable pregnancy outcome [6], [8], [9].

There are three treatment options for GD: medical therapy with antithyroid drugs (ATDs), radioactive iodine (^131^I) and surgery [6], [10]. Radioactive iodine therapy of maternal GD is contraindicated during pregnancy [6], [11]. Surgery is associated with a risk of miscarriage [11]. Thus ATDs are the preferred treatment for GD during pregnancy [12].

In the US, propylthiouracil (PTU) and methimazole (MMI) are the two ATDs available. In some countries, carbimazole, which is converted to MMI, is available [13]. MMI is 20-fold more potent than PTU and has a longer half-life [2], [13]. Both medications control the hyperthyroid state [2], [14], [15] and cross the placenta similarly [16]. Fetal concentrations of PTU and MMI are 30–80% of maternal levels [17]. The structure-activity relationships of these compounds has been recently elucidated [18].

Reports suggest a relationship between congenital anomalies (choanal atresia, aplasia cutis, esophageal atresia, facial abnormalities, hypothelia, cardiovascular defects) and prenatal exposure to MMI, a constellation of features termed MMI embryopathy [19], [20], [21], [22]. Large cohort studies, however, have found birth defects associated with maternal GD to be attributed to the hyperthyroid state rather than to the ATDs themselves [23], [24], [25].

Because of the few number of reports of fetal anomalies associated with maternal use of PTU during pregnancy as compared to MMI, PTU has been assumed to have a more favorable teratogenic profile and is therefore recommended for use during pregnancy [6], [26]. There have, however, been a small number of birth defects reported in association with prenatal PTU use since its introduction in 1947 [27], [28], [29], [30], [31].

Recently a hepatotoxicity risk of PTU has been uncovered [32]. Thus to minimize risks to the mother and fetus, it has been suggested that PTU use be restricted to the 1^st^ trimester of pregnancy and changed to MMI thereafter [8], [12]. This recommendation, though, is based on limited data [8].

Other adverse effects of PTU use during pregnancy have also been observed including agranulocytosis, and skin rash and vasculitis [33]. Iatrogenic fetal goitrous hypothyroidism can result from overtreatment of graves during pregnancy [34]. Iatrogenic fetal goitrous hypothyroidism can affect the normal fetal development and cause several developmental anomalies including polyhydramnios, hyperextension of the fetal neck, intrauterine growth restriction, fetal hydrops and delayed bone development [35], [36].

Despite widespread ATD use in pregnancy, formal studies of ATD teratogenic effects have yet to be performed in humans [37]. There has been some investigation of the effects of ATDs in animal models. Studies of E9.5 to E11.5 rat embryos cultured with high doses of MMI revealed abnormal head morphology and absence of neural tube closure [38]. Treatment of rabbits and guinea pigs with PTU after embryogenesis led to thyroid enlargement but no congenital anomalies [39]. Treatment of rats, mice and rabbits after embryogenesis did not cause congenital anomalies [40], [41], [42].

To begin to address the limitations in our understanding of the teratogenic potential of ATDs, we examined the effects of PTU and MMI exposure during embryogenesis in mice.

## Materials and Methods

### Mice

All animal studies were approved by the Yale University Institutional Animal Care and Use Committee under protocol #2009-11315. Mice were used for study, as they are a validated model of teratogenicity investigation [43]. C57BL/6 mice (Charles River Laboratories, Wilmington, MA, USA) were housed in a temperature-controlled room with a 12-hour light-dark cycle and access to food and water ad libitum. For breeding, males and females were paired overnight. The day a vaginal plug was observed was designated embryonic day (E) 0.5.

### Drug treatments

Drugs were administered orally via gavage in 0.5 ml of water. The PTU (Sigma Aldrich; St. Louis, MO) doses used were 1, 5, 10, 25, 50 and 100 mg/kg. In preliminary studies, the PTU dose of 200 mg/kg was found to be lethal. The MMI (Sigma Aldrich) doses used were 1, 4, 10 and 20 mg/kg. Controls were administered vehicle (distilled water). Doses were given each day between 12:00 to 15:00 hrs. To span critical periods of embryogenesis [44], [45], dams were initially treated each day between E7.5 and E9.5. To investigate effects at PTU exposure on earlier stages of development, pregnant mice were treated with 100 mg/kg of PTU or 20 mg/kg of MMI each day from E3.5 to E7.5. Physical observations of the treatment groups indicated that ATD treatment did not result in any observable effects on maternal behavior. When maternal weights were assessed at the end of the treatment periods, difference in the maternal body weight among the groups was not observed.

### Necropsy and histopathology

After drug treatments, embryos were examined at E10.5 or E18.5. At E10.5, dams were euthanized by CO_2_ asphyxiation. Embryos were removed from the uteri and rinsed in Dulbecco's phosphate buffered saline (D-PBS, InVitrogen, Carlsbad, CA). Embryos were photographed using an Olympus C-5060 camera (Tokyo, Japan) attached to a Ziess Stemi 2000C dissecting microscope (Oberkochen, German). Photographic images were used to measure the crown rump lengths of embryos. Gross evaluation of embryos included assessment of viability and the presence or absence of gross malformations. Embryos were considered alive if their hearts were beating. Dead embryos (no heart beat) were excluded from analysis.

At E18.5, pregnant dams were euthanized by CO_2_ followed by cervical dislocation and exsanguination by cardiac puncture. Embryos and placentas were harvested from the uterus, weighed, fixed in Bouin's fixative (Ricca Chemical Corporation, Arlington TX), embedded in paraffin, sectioned at 5 µm, and stained with Masson's Trichrome (head sections only) by routine methods [46]. Masson's Trichrome-stained coronal sections of the head were examined for craniofacial and neurologic defects by light microscopy and digital image recorded using a Zeiss AxioImager A1 Axioskop microscope, AxoCam MRc55 camera and AxioVision 4.7.1 imaging software (Carl Zeiss Micro Imaging, Inc. Thornwood, NY) where the reviewer was blind to experimental manipulation. Images were generated in Adobe® Photoshop® (Adobe Systems Incorporated, San Jose, California).

### MicroCT imaging

Embryos were collected for microCT scanning at E10.5 and E18.5, stained in 1% iodine in ethanol for at least 24 hours, and rinsed in 70% ethanol. Scans were performed in 70% ethanol in a mCT-35 scanner (Scanco, Bruttisellen, Switzerland) at an isometric voxel size of 6 µm, peak energy of 45 kVp, and an integration time of 800 ms. Images were analyzed using Microview software v2.0 (GE Healthcare).

### Gene expression analysis

Total RNA was extracted from whole embryos according to the manufacturer's instructions and purified with the RNeasy Kit (Qiagen, Inc.). RNA concentrations were obtained from absorbance at 260 nm (A260). RNA quality was determined based on an A260/A280 ratio. The W.M. Keck Facility at Yale University performed microarray analysis. Total RNA samples were hybridized to Illumina Sentrix BeadChip array (Mouse WG-6 v2; Illumina Inc., San Diego, CA) following preparation using the Illumina TotalPrep RNA Amplification kit (Applied Biosystems; Austin, TX). GenomeStudio software (Illumina Inc.) was used to assess changes in gene expression between controls and treatment groups. Gene pathway analysis was performed using Metacore software (Genego Pathway Analysis; St. Joseph, MI).

### Hormone measurements

Plasma thyroxine (T4) levels were measured by radioimmunoassay (RIA) using a Mouse/Rat Thyroxine ELISA kit (GenWay Biotech, Inc, San Diego, CA). Serum samples were obtained at E10.5 from the pregnant dams that were treated with PTU (10, 25, 50, 100 mg/kg), MMI (10, 20 mg/kg) or vehicle from E7.5 to E9.5.

### Statistical analysis

Statistical analysis was performed with repeated measures analysis using mixed models with Tukey's approach for multiple comparisons, and with generalized estimating equations (GEE), by Statistical Analysis Software (SAS). Fisher's exact test analysis was also performed. Data are presented as mean ± SEM. T4 hormone levels between treatment groups were compared using one-way ANOVA.

## Results

### Effects of PTU and MMI on embryonic development

We first examined the effects of PTU and MMI during a critical period of embryogenesis. Dams were treated from E7.5 to E9.5 with 1, 5, 10, 25, 50 or 100 mg/kg of PTU, or with 1, 4, 10 or 20 mg/kg of MMI, and embryos were examined at E10.5. Per standard protocols used to assess drug teratogenic potential, doses used were multiples of standard human doses [47].

Data were obtained from a total of 713, E10.5 embryos from 64 dams. PTU and MMI treated-embryos did not differ in size among treatment doses, but were larger than control embryos (mean crown-rump length PTU 4.42 mm; MMI 4.41 mm, control 4.12 mm, p<0.001, Table 1). No significant difference in T4 hormone levels was observed in dams exposed to any doses of PTU, MMI or vehicle. At that highest doses used, T4 levels were: PTU: 3.72±0.4 µ/dl, n = 20; MMI, 2.3±0.18 µg/dl, n = 4; vehicle, 3.3±0.26 µg/dl, n = 12P>0.05).

**Table 1:** Crown-rump length and viability of E10.5 murine embryos.

Drug	Dose (mg/kg)	Embryo (n)	Crown-rump length (mm) Mean (SE)	P value^1^	Dead (%)
**PTU (n = 413)**	1	45	4.33 (0.19)		4 (8.9)
	5	47	4.36 (0.09)		2 (4.3)
	10	37	4.38 (0.09)	0.0082	1 (2.7)
	25	81	4.49 (0.07)		8 (9.9)
	50	58	4.37 (0.08)		5 (8.6)
	100	145	4.64 (0.06)		5 (3.4)
**MMI (n = 146)**	1	36	4.56 (0.09)	0.0015	1 (2.8)
	4	46	4.40 (0.09)		6 (13)
	10	24	4.03 (0.11)		0 (0)
	20	40	4.56 (0.10)		8 (20)
**Vehicle (n = 145)**	-	154	4.12 (0.06)		9 (5.8)

1 Mixed model with repeated measure analysis (adjusted by Tukey approach for multiple comparisons) between PTU vs. vehicle and MMI vs. vehicle.

SE, Standard Error.

**Figure 1:**
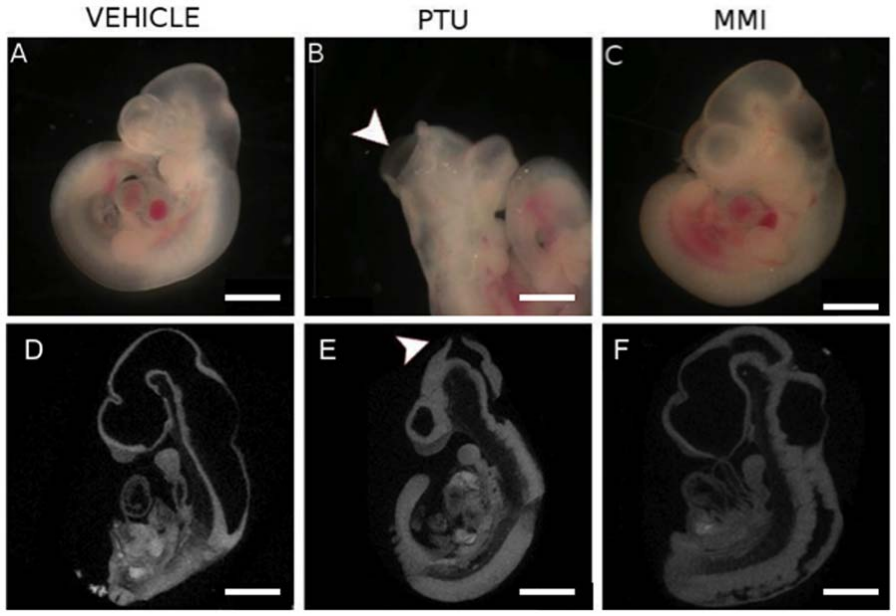
PTU exposure during embryogenesis induces cranial defects. Representative images of E10.5 embryos treated with (A, D) vehicle, (B, E) PTU (100 mg/kg) or (C, F) MMI (20 mg/kg) illustrate un-fused cephalic neural folds in PTU-treated embryos (arrowhead). (D–F) MicroCT scans showing sagittal 3D images of E10.5 whole embryos with arrowhead identifying unfused neural folds defect. Scale bars = 0.5 mm.

Next, we evaluated for the presence or absence of gross malformations (Table 2). Of those observed, cranial and cardiac structural defects were the most common. Cranial defects were more common among the PTU-exposed embryos than those exposed to MMI (p≤0.17 vs. vehicle, Fisher's exact test) or vehicle (p≤0.0002 vs. vehicle, Fisher's exact test; Fig. 1. Table 2). Cranial defects were greatest at the highest PTU dose of 100 mg/kg (12.9%, p≤0.0001 vs. vehicle, Fisher's exact test).

**Table 2:** Structural defects in E10.5 murine embryos.

Drug	Dose mg/kg	# of Embryos	Type of Defects			Total Sum of Defects		
			Head folds not fused	Peri-cardial blood	Other defects	Embryo Defects	Odds Ratio	p-value
			n (%)	n (%)	n (%)	n (%)	OR (95% CI)	
PTU	1	41	2 (4.8)	2 (4.4)	0 (0.0)	4 (9.7)	1.6 (0.5, 5.2)	0.24
	5	45	1 (2.2)	2 (4.2)	2 (4.2)	5 (11.1)	1.8 (0.6, 5.6)	0.15
	10	36	4 (11.1)	3 (8.1)	1 (2.7)	8 (22.2)	4.1 (1.5, 11.3)	0.0019
	25	73	3 (4.1)	6 (9)	3 (1.4)	12 (16.4)	2.8 (1.2, 6.9)	0.009
	50	53	3 (5.6)	6 (8)	0 (0)	9 (17)	2.9 (1.1, 7.7)	0.011
	100	140	18 (12.9)	13 (9.2)	5 (1.4)	36 (25.7)	4.9 (2.4, 10.5)	<0.0001
MMI	1	35	2 (5.7)	0 (0)	1 (2.5)	3 (8.6)	1.4 (0.4, 5.2)	0.33
	4	40	1 (2.5)	2 (5)	0 (0)	3 (7.5)	1.7 (0.3, 4.5)	0.41
	10	24	0 (0)	2 (8.3)	1 (4.2)	3 (12.5)	2.1 (0.5, 8.1)	0.15
	20	32	2 (6.2)	3 (9.3)	0 (0)	5 (15.6)	2.7 (0.8, 8.4)	0.04
Vehicle		154	3 (2)	7 (4.8)	0 (0)	10 (6.9)		

Includes live embryos only.

Chi-square \ test.

**Figure 2:**
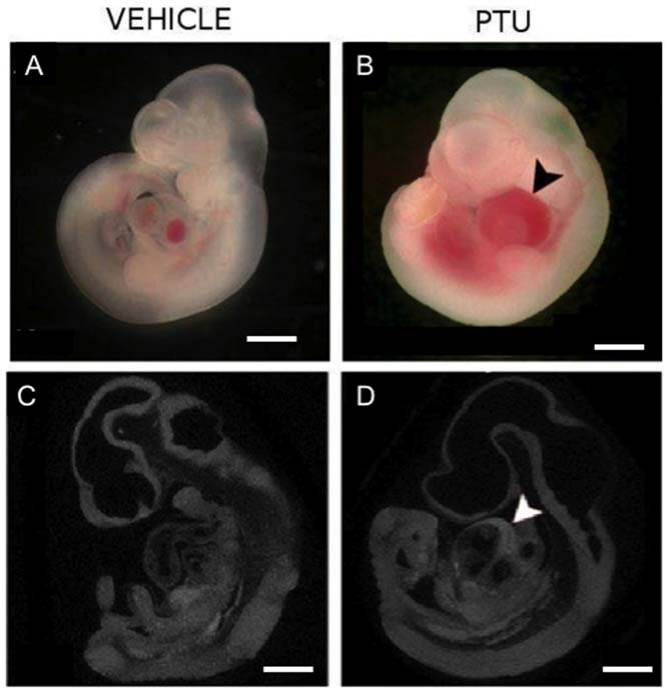
PTU exposure during embryogenesis induces blood in the pericardium. Representative images of E10.5 embryos treated with (A, C) vehicle and (B, D) PTU (100 mg/kg) showing blood in the pericardial sac of PTU-treated embryo (arrowhead). (C, D) MicroCT scans showing sagittal 3D images of whole embryos. Scale bars = 0.5 mm.

Blood in the pericardial sac, which is a feature indicative of abnormal cardiac function and/or abnormal vasculature [48], was observed more frequently in PTU-treated than MMI-treated or vehicle-treated embryos (Fig. 2. Table 2). Other abnormalities observed included growth-retardation, abnormal head shape (without head defects) and spinal defects. These abnormalities were observed more frequently in PTU-treated than vehicle-treated embryos (p≤0.01, Fisher's exact test), but not in MMI-treated vs. vehicle-treated embryos (p = 0.06, Fisher's exact test) (Table 2).

Overall, the PTU doses of 10, 25, 50, 100 mg/kg induced embryo malformations at rates higher than control (Table 2). Only at the highest dose of MMI (20 mg/kg), there was a slight increase in the malformation rate compared to vehicle and the defects mainly consisted of blood in the pericardial sac (p = 0.04; Table 2).

### PTU-mediated changes in gene expression

To investigate potential mechanisms associated with unfused head folds in the PTU-exposed embryos, microarray analysis was performed. Because we did not observed defects with MMI, only affects of PTU were investigated.

**Figure 3:**
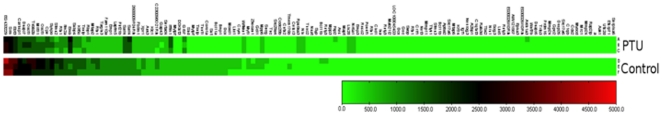
Heat map showing the differential gene expression of 139 genes between PTU and vehicle treated groups. Heat map generated in GenomeStudio compares the average detection signals of genes between PTU and control groups. Total RNA was collected at E10.5 from whole-embryos treated between E7.5–E9.5,. Three PTU and three control RNA samples were tested and genes with a significant p-value (p<0.001) were displayed. Scale bar indicates detection signal.

E10.5 embryos with unfused cephalic neural folds from dams that had been treated with 100 mg/kg of PTU from E7.5 to E9.5 were compared with vehicle controls. A total of 134 differentially expressed genes were identified in the PTU-treated embryos. Nineteen genes had increased expression and 115 had decreased expression. A complete list of genes is presented in Table S1) and a representative heat map is presented in Figure 3.

**Figure 4:**
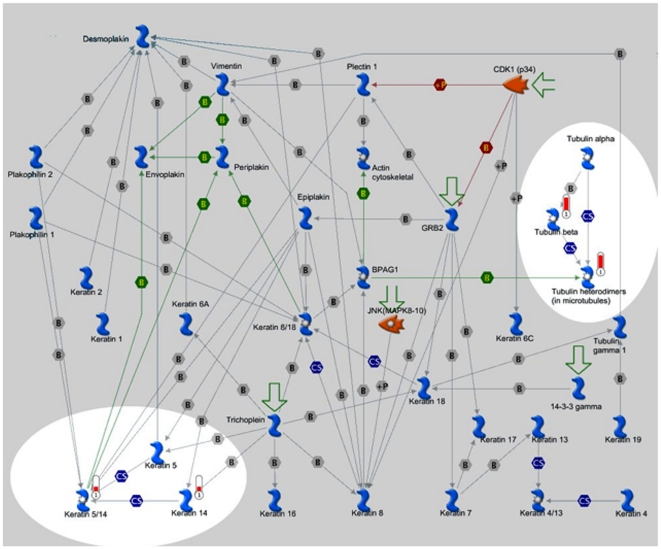
Expression of Genes in the Cytoskeleton remodeling and keratin filament pathways were altered by PTU treatment. Cytoskeleton structure in most eukaryotic cells consists of three distinct interconnected, filament systems: Actin filaments, Microtubules and intermediate filaments. Cell assembly is integrated by the network of intermediate filaments (IFs) and by their interactions with other cytoskeleton structural elements defining cytoarchitecture and cytodynamics. The IF network is critically involved in cell shape control and imparts intracellular mechanical strength. The experimental data are visualized on the map in red (increased expression or up-regulation) histogram. The height of the histogram corresponds to the relative expression value for a particular gene/protein.

Metacore-GeneGo pathway analysis was used to identify potentially disrupted molecular pathways (Table 3). The most significantly disrupted pathways were those associated with cytoskeleton remodeling and keratin filaments (Figure 4).

**Table 3:** Molecular pathways affected by exposure to PTU in E10.5 embryos.

#	Pathway	# of Genes	Fold- change Microarray	p-value
1	Cytoskeleton remodeling keratin filaments	2 of 36	2.8	0.001
2	Transport_RAB1A regulation pathway	1 of 12	1.7	0.019
3	Transport_RAB3 regulation pathway	1 of 14	1.7	0.02
4	Development_ERK5 in cell proliferation and neuronal survival	1 of 23	1.4	0.04
5	Development_GDNF signaling	1 of 24	1.4	0.04
6	Development_Mu-type opioid receptor signaling via Beta-arrestin	1 of 24	1.4	0.04
7	Proteolysis_Role of Parkin in the Ubiquitin-Proteasomal Pathway	1 of 24	1.4	0.04
8	Cytoskeleton remodeling_Neurofilaments	1 of 25	1.4	0.04
9	Development_SSTR1 in regulation of cell proliferation and migration	1 of 29	1.3	0.045
10	Cell adhesion_Gap junctions	1 of 30	1.3	0.047

### Effects of PTU and MMI exposure during embryogenesis on near term-embryos

**Figure 5:**
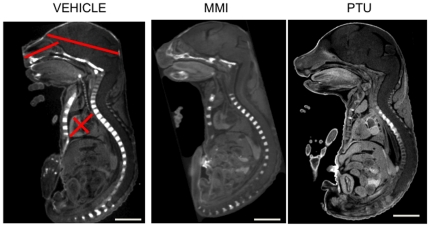
PTU exposure during embryogenesis and effects in E18.5 embryos. Embryos treated *in utero* from E7.5–E9.5 were analyzed at E18.5. Representative MicroCT scans showing sagittal 3D images of whole embryos indicate no differences between the treatment groups of vehicle, PTU, and MMI. Left panel shows location of morphometic measurements (a. Snout: upper lip to soft palate. b. Fronto-occipital: frontal to occipital lobes. c. Aorta-apex diameter: aortic valve to left ventricular. d. Biventricular diameter: right to left ventricle). Scale bars = 5 mm.

**Figure 6:**
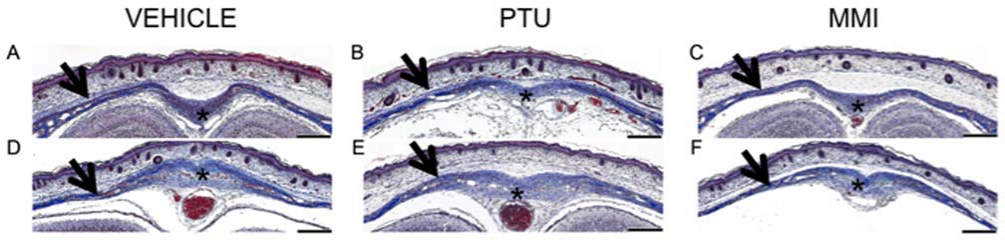
Representative histopathology of E18.5 calveria from vehicle, PTU and MMI treated mice. Histopathologic analysis of coronal sections through the head of E18.5 embryos from dams treated with (A, D) vehicle, (B, E) PTU (100 mg/kg), or (C, F) MMI (20 mg/kg) from E7.5 to E9.5 did not reveal any significant morphologic changes in the bones of the calveria (arrowheads) or the connective tissue stroma within the (A, B, C) frontal or (D, E, F) parietal sutures when compared to the skulls of control embryos. Representative sections from (A, B, C) rostral dorsal frontal bone at the level of the nasal cortex, and (D, E, F) caudal dorsal parietal at the level of the ears. Scale bars = 200 µm. Masson's Trichrome stained sections are shown.

To determine if the abnormalities observed at E10.5 persisted, embryos were treated with ATDs from E7.5 to E9.5 and examined at E18.5. Embryo weights at E18.5 were similar among the different groups (PTU, 1.16±0.03 g, n = 35; MMI, 1.16±0.05 g, n = 14; and vehicle, 1.18±0.03 g, n = 3). No gross malformations were evident in either ATD group at E18.5 (Figure 5). To assess if craniofacial or heart structural development was affected, snout, head and heart size were assessed using MicroCT. Analysis of MicroCT images did not reveal any differences among comparative measurements of snout length, brain or heart size (Fig. 5; Table 4). Histopathological analysis of coronal sections through the head also did not reveal differences among the PTU, MMI and vehicle groups (Fig. 6).

**Table 4:** Morphometic Analysis of Embryos at E18.5 based on MicroCT images.

	Vehicle	PTU	MMI	p-value^1^
Parameter (mm)	Mean(SEM)	Mean(SEM)	Mean(SEM)	
**Snout length**	5.13	4.97	5..27	0.28
	(0.10)	(0.07)	(0.12)	
**Brain Fronto-**	9.27	9.97	8.61	0.09
**occipital diameter**	(0.22)	(0.20)	(0.23)	
**Aorta-apex diameter**	3.16	3.11	2.86	0.08
	(0.07)	(0.08)	(0.15)	
**Bi-ventricular**	2.52	2.65	2.45	0.15
**diameter**	(0.05)	(0.09)	(0.09)	

1 ANOVA.

### Effects of early exposure to PTU during embryogenesis on heart development

We next assessed effects of PTU and MMI at even earlier stages of development to investigate if abnormal looping and/or situs inversus could be elicited with ATD exposure. Dams were treated with 100 mg/kg of PTU or 20 mg/kg of MMI from E3.5 to E7.5. No significant differences in craniofacial defect rates at E10.5 were observed between control and ATD groups. Situs defects were not observed either in control or ATD groups.

Early exposure (E3.5 to E7.5) in mice though, resulted in growth retardation in the PTU-treated group (n = 52) with a mean crown-rump length of 4.24±0.08 mm vs. the vehicle-treated group (n = 67) with a mean crown-rump length of 4.89±0.06 mm (p<0.0001). No significant difference in crown-rump length was observed between MMI (mean crown-rump length 4.74±0.06 mm) and control group (p>0.05).

## Discussion

We show that exposure to PTU during critical periods of embryogenesis causes structural anomalies in mice. PTU-treated embryos exhibited delayed fusion of head neural folds and hemorrhagic pericardial sacs. Cranial defects consisted of unfused cephalic neural folds, a process normally completed by E9 in mouse embryos [44].These results suggest that the notion that PTU is not teratogenic is incorrect [12].

It is important to note that the observed effects cannot be attributable to the possibility of hypothyroidism. The morphogenesis of fetal thyroid cells begins at E 8.5 but thyroid gland is not known to become functional to produce thyroid hormone until E 16.5 [49], well after our treatment period. Prior to the fetal thyroid hormone production, the fetus is dependent on the transplacental transport of thyroxine [50], [51]. When we assessed T4 levels of the dams that were exposed to antithyroid drugs, they were similar to the control group. Thus it is very unlikely that teratogenicity was the result of a hypothyroid state in the dams or fetus.

Previous animal studies on potential effects of PTU or MMI are limited [13]. Thyroid enlargement and hypothyroidism have been observed in neonates treated with PTU and MMI *in utero*, but the rate of major congenital anomalies was not increased [13], [52]. MMI reportedly caused abnormal head morphology and absence of neural tube closure of rat embryos cultured from E9.5 to 11.5, however, the concentration at which MMI disturbed rat embryogenesis was very high [13], [38].

Despite several reports of an association of birth defects and prenatal use of MMI [19], [20], [21], [22], this association remains unclear [8]. Momotani et al. reported that among 643 infants of mothers with Graves' disease, uncontrolled maternal hyperthyroidism was associated with congenital malformations, whereas MMI treatment was not [24]. Van Dijke and co-workers found no increased birth defect rates in the setting of MMI use on review of 49,091 records [25]. Wing et al. reached similar observations in a review of 36 infants exposed to MMI [15], and a cohort of 241 infants born to MMI-treated woman, had no increased in birth defects as reported by Di Gianantonio [23]. Our observations are consistent with these clinical findings, as we did not see MMI-associated birth defects.

In a recent report, congenital cardiac defects were associated with PTU in an analysis that included over 18,000 congenital malformations and 127 infants exposed to ATD during the first trimester, 8 of whom were exposed to PTU [22]. In our study, we did not observe situs abnormalities in the embryos exposed to PTU during early embryogenesis. PTU embryos, though, were twice as likely to have blood in the pericardial sacs, which is a feature indicative of embryo cardiac dysfunction [48].

A recent epidemiological study on birth defects associated with the treatment of hyperthyroidism suggested that exposure to PTU was associated with situs inversus and cardiac outflow tract defects [22]. In murine embryos, axial determination begins with rightward looping at stage E6 [53]. In our studies, we did not observe altered cardiac looping from PTU or MMI exposure. These discrepant observations may reflect species related differences or difference in the timing and doses of antithyroid drugs in clinical practice vs. the treatment paradigms used here. At present, we are unaware of studies that had described neural tube defects associated with PTU use. However, neural tube defects have been reported to the US FDA associated with PTU use (FDA MedWatch). Potential teratogenic mechanisms associated with delayed skull closure may be related to altered differential gene expression patterns that involve cytoskeleton remodeling, which in turn define cell cytoarchitecture and cytodynamics. In our study, PTU altered cytoskeleton remodeling and keratin filaments signaling pathways. These pathway components play a role in cytoskeleton organization, epithelium morphogenesis and neuronal survival (Fig. 4). Major skull defects were not found in near-term embryos in any of the experimental groups. This indicates that PTU causes delayed neural tube closure. It is also possible that there is resorption of malformed embryo, an issue that is currently under investigation by our group. In summary, we observed that PTU exposure during embryogenesis was associated with delayed neural tube closure and cardiac abnormalities. In contrast, we did not observe structural defects associated with MMI exposure except at the higher doses and majority of these were mainly cardiac defects.

These observations dispute the notion that PTU is not teratogenic. Further human epidemiological studies of this important issue are needed.

## Supporting Information

Table S1 Genes differentially expressed in PTU-treated E10.5 embryo.
(DOC)
