# Supplementary material for: Propylthiouracil Is Teratogenic in Murine Embryos
Source: PLoS One. 2012 Apr 18;7(4):e35213. doi: 10.1371/journal.pone.0035213 (PMC3329451; doi:10.1371/journal.pone.0035213)
Supplement: Table S1 — Genes differentially expressed in PTU-treated E10.5 embryo. (DOC) [file pone.0035213.s001.doc]

**Supplementary data**

**Table S1: Genes differentially expressed in PTU-treated E10.5 embryos**

|  | **ProbeID**  **Illumina®** | **Gene** | | **Fold Change** | **P-Value**  **<0.05** |
| --- | --- | --- | --- | --- | --- |
| **Genes up-regulated in PTU treated embryos** | | | | | |
| 1 | 2120500 | ETSRP71 | | 2.63 | 0.001 |
| 2 | 6860168 | ALDH1A3 | | 2.53 | 0.020 |
| 3 | 5960735 | WFIKKN1 | | 2.39 | 0.000 |
| 4 | 6040066 | CDX2 | | 2.35 | 0.001 |
| 5 | 1710747 | NR6A1 | | 2.03 | 0.000 |
| 6 | 6290445 | LIN28 | | 1.96 | 0.018 |
| 7 | 6760673 | MYOCD | | 1.96 | 0.002 |
| 8 | 4010019 | IFITM1 | | 1.81 | 0.002 |
| 9 | 7330551 | SALL4 | | 1.71 | 0.000 |
| 10 | 2320315 | SALL4 | | 1.70 | 0.046 |
| 11 | 780348 | GRB7 | | 1.62 | 0.002 |
| 12 | 4280056 | TRIB3 | | 1.59 | 0.032 |
| 13 | 240736 | RPS6KA3 | | 1.57 | 0.004 |
| 14 | 270341 | EMB | | 1.56 | 0.038 |
| 15 | 5690754 | CHD1 | | 1.51 | 0.025 |
| 16 | 3710594 | PKP2 | | 1.48 | 0.034 |
| 17 | 4640397 | NIN | | 1.48 | 0.025 |
| 18 | 2810673 | MPP6 | | 1.48 | 0.046 |
| 19 | 6760180 | SRM | | 1.40 | 0.027 |
| **Genes down-regulated in PTU-treated embryos** | | | | | |
| 1 | 6660403 | | SERPINA6 | 13.28 | 0.030 |
| 2 | 2060725 | | UTS2D | 5.11 | 0.007 |
| 3 | 460692 | | BCL11A | 3.82 | 0.000 |
| 4 | 6110008 | | BCL11B | 3.61 | 0.017 |
| 5 | 4780019 | | GSC | 3.57 | 0.000 |
| 6 | 6840709 | | SLC17A6 | 3.36 | 0.019 |
| 7 | 7200021 | | SNCG | 3.25 | 0.000 |
| 8 | 1780433 | | SLC17A6 | 3.12 | 0.007 |
| 9 | 3060328 | | LHX1 | 3.05 | 0.001 |
| 10 | 7650475 | | BCL11B | 2.85 | 0.027 |
| 11 | 1500575 | | AAK1 | 2.79 | 0.046 |
| 12 | 5050471 | | NRN1 | 2.78 | 0.028 |
| 13 | 580332 | | C1QB | 2.78 | 0.000 |
| 14 | 1110255 | | RAP2IP | 2.72 | 0.007 |
| 15 | 580037 | | MMP24 | 2.60 | 0.024 |
| 16 | 5890291 | | A | 2.51 | 0.022 |
| 17 | 3130768 | | STMN3 | 2.47 | 0.001 |
| 18 | 4730220 | | MRC1 | 2.33 | 0.009 |
| 19 | 3710209 | | A2BP1 | 2.28 | 0.003 |
| 20 | 2260136 | | DCX | 2.28 | 0.001 |
| 21 | 6280240 | | CRYM | 2.26 | 0.017 |
| 22 | 130349 | | MYLPF | 2.25 | 0.000 |
| 23 | 2360152 | | MMP24 | 2.24 | 0.001 |
| 24 | 3830220 | | AW121567 | 2.22 | 0.041 |
| 25 | 6940209 | | LOC100045403 | 2.18 | 0.000 |
| 26 | 1070239 | | PRDM16 | 2.16 | 0.003 |
| 27 | 3780450 | | CRLF1 | 2.15 | 0.000 |
| 28 | 5860093 | | GPC2 | 2.15 | 0.009 |
| 29 | 1090504 | | MAPT | 2.12 | 0.016 |
| 30 | 870086 | | MYT1 | 2.12 | 0.000 |
| 31 | 3870095 | | STMN2 | 2.10 | 0.000 |
| 32 | 4810288 | | RTN1 | 2.10 | 0.009 |
| 33 | 4860132 | | RUFY3 | 2.05 | 0.017 |
| 34 | 2000647 | | IGF1 | 2.04 | 0.000 |
| 35 | 4610431 | | TTYH1 | 2.03 | 0.000 |
| 36 | 1440088 | | WNT7B | 2.02 | 0.004 |
| 37 | 3870471 | | DCX | 2.01 | 0.000 |
| 38 | 2470626 | | RUFY3 | 2.00 | 0.027 |
| 39 | 4040154 | | C230098O21RIK | 1.98 | 0.000 |
| 40 | 2070594 | | DNAJC6 | 1.98 | 0.019 |
| 41 | 430768 | | GDI1 | 1.98 | 0.000 |
| 42 | 3830605 | | CELSR3 | 1.96 | 0.000 |
| 43 | 270671 | | C1QTNF3 | 1.91 | 0.004 |
| 44 | 3370279 | | E030026I10RIK | 1.91 | 0.007 |
| 45 | 4150278 | | INA | 1.91 | 0.000 |
| 46 | 5340301 | | RUFY3 | 1.89 | 0.002 |
| 47 | 620064 | | TNNT1 | 1.89 | 0.005 |
| 48 | 460075 | | DUSP26 | 1.88 | 0.020 |
| 49 | 990315 | | RTN1 | 1.85 | 0.004 |
| 50 | 4730474 | | TAGLN3 | 1.85 | 0.000 |
| 51 | 6220632 | | MTAP2 | 1.83 | 0.000 |
| 52 | 990392 | | SYP | 1.83 | 0.000 |
| 53 | 6760050 | | EPHA3 | 1.82 | 0.022 |
| 54 | 670059 | | POU4F1 | 1.82 | 0.000 |
| 55 | 2630079 | | SCG3 | 1.81 | 0.032 |
| 56 | 270681 | | E030026I10RIK | 1.80 | 0.015 |
| 57 | 1470619 | | KRT14 | 1.80 | 0.008 |
| 58 | 5270520 | | SVOP | 1.79 | 0.002 |
| 59 | 6770615 | | MYLPF | 1.78 | 0.000 |
| 60 | 6250193 | | TXNIP | 1.77 | 0.000 |
| 61 | 6380255 | | RTN1 | 1.76 | 0.000 |
| 62 | 3520546 | | EMP3 | 1.76 | 0.001 |
| 63 | 3420110 | | TNNC2 | 1.76 | 0.004 |
| 64 | 670367 | | TBX2 | 1.75 | 0.028 |
| 65 | 4050347 | | NEUROG3 | 1.74 | 0.015 |
| 66 | 1470278 | | RAB3D | 1.73 | 0.034 |
| 67 | 2370687 | | RTN1 | 1.72 | 0.000 |
| 68 | 5860315 | | MMP11 | 1.71 | 0.009 |
| 69 | 1500463 | | CORO1A | 1.70 | 0.000 |
| 70 | 6100471 | | RUSC2 | 1.70 | 0.001 |
| 71 | 2370274 | | LHX5 | 1.69 | 0.035 |
| 72 | 650184 | | 2600009P04RIK | 1.68 | 0.003 |
| 73 | 5570315 | | APBA2 | 1.67 | 0.022 |
| 74 | 7210440 | | MLLT11 | 1.67 | 0.005 |
| 75 | 5670598 | | MTAP6 | 1.65 | 0.025 |
| 76 | 3450180 | | TMEM176B | 1.64 | 0.008 |
| 77 | 580519 | | MAB21L2 | 1.63 | 0.009 |
| 78 | 5900551 | | PHF21B | 1.61 | 0.000 |
| 79 | 3450170 | | CCDC28B | 1.61 | 0.007 |
| 80 | 5670634 | | SYNPO | 1.61 | 0.008 |
| 81 | 10195 | | NEF3 | 1.60 | 0.034 |
| 82 | 3420500 | | OXCT1 | 1.60 | 0.001 |
| 83 | 4670193 | | SST | 1.59 | 0.007 |
| 84 | 4390538 | | ITM2A | 1.58 | 0.000 |
| 85 | 6370148 | | CNTN2 | 1.57 | 0.005 |
| 86 | 4200193 | | AES | 1.57 | 0.017 |
| 87 | 3460670 | | OXCT1 | 1.55 | 0.000 |
| 88 | 1510671 | | MPP3 | 1.55 | 0.015 |
| 89 | 1030482 | | SLC29A4 | 1.54 | 0.007 |
| 90 | 6130468 | | KLC1 | 1.54 | 0.001 |
| 91 | 3190639 | | KIF5C | 1.54 | 0.001 |
| 92 | 1030475 | | SSBP4 | 1.53 | 0.007 |
| 93 | 2360541 | | NNAT | 1.53 | 0.023 |
| 94 | 3450524 | | ZKSCAN1 | 1.53 | 0.022 |
| 95 | 7380364 | | CARHSP1 | 1.52 | 0.001 |
| 96 | 650561 | | TUBB2B | 1.51 | 0.019 |
| 97 | 70341 | | TXNIP | 1.51 | 0.001 |
| 98 | 6860609 | | RBP1 | 1.51 | 0.001 |
| 99 | 70646 | | MOXD1 | 1.50 | 0.013 |
| 100 | 940438 | | LAPTM5 | 1.50 | 0.008 |
| 101 | 270324 | | TNC | 1.49 | 0.035 |
| 102 | 4050477 | | D0KIST3 | 1.47 | 0.020 |
| 103 | 380639 | | SERTAD4 | 1.45 | 0.018 |
| 104 | 1070184 | | XPR1 | 1.44 | 0.050 |
| 105 | 460110 | | GAP43 | 1.44 | 0.022 |
| 106 | 6580240 | | BMP1 | 1.44 | 0.040 |
| 107 | 2690743 | | SHD | 1.44 | 0.028 |
| 108 | 1090228 | | FAM110A | 1.43 | 0.013 |
| 109 | 4260195 | | FBLIM1 | 1.42 | 0.021 |
| 110 | 4290630 | | ANK3 | 1.42 | 0.027 |
| 111 | 6770497 | | EBF3 | 1.42 | 0.020 |
| 112 | 240259 | | EG433229 | 1.41 | 0.015 |
| 113 | 7160167 | | EMP1 | 1.40 | 0.030 |
| 114 | 3390243 | | CPE | 1.37 | 0.041 |
| 115 | 3840075 | | DNAJC7 | 1.37 | 0.041 |
